# Application of AI in cardiovascular multimodality imaging

**DOI:** 10.1016/j.heliyon.2022.e10872

**Published:** 2022-10-05

**Authors:** Giuseppe Muscogiuri, Valentina Volpato, Riccardo Cau, Mattia Chiesa, Luca Saba, Marco Guglielmo, Alberto Senatieri, Gregorio Chierchia, Gianluca Pontone, Serena Dell’Aversana, U. Joseph Schoepf, Mason G. Andrews, Paolo Basile, Andrea Igoren Guaricci, Paolo Marra, Denisa Muraru, Luigi P. Badano, Sandro Sironi

**Affiliations:** a Department of Radiology, Istituto Auxologico Italiano IRCCS, San Luca Hospital, Italy; b School of Medicine, University of Milano-Bicocca, Milan, Italy; c Department of Cardiac, Neurological and Metabolic Sciences, San Luca Hospital, Istituto Auxologico Italiano IRCCS, Milan, Italy; d Department of Radiology, Azienda Ospedaliero Universitaria (A.O.U.), di Cagliari, Polo di Monserrato, Cagliari, Italy; e Centro Cardiologico Monzino IRCCS, Milan, Italy; f Department of Cardiology, Division of Heart and Lungs, Utrecht University, Utrecht University Medical Center, Utrecht, the Netherlands; g Department of Radiology, Ospedale S. Maria Delle Grazie - ASL Napoli 2 Nord, Pozzuoli, Italy; h Division of Cardiovascular Imaging, Department of Radiology and Radiological Science, Medical University of South Carolina, Ashley River Tower, 25 Courtenay Dr., Charleston, SC, USA; i University Cardiology Unit, Department of Emergency and Organ Transplantation, University of Bari, Bari, Italy; j IRCCS Ospedale Galeazzi - Sant'Ambrogio, University Cardiology Department, Milan, Italy; k Department of Radiology, ASST Papa Giovanni XXIII, 24127 Bergamo, Italy

**Keywords:** Cardiac computed tomography angiography, Cardiac magnetic resonance, echocardiography, Artificial intelligence, Coronary plaque, Late gadolinium enhancement

## Abstract

Technical advances in artificial intelligence (AI) in cardiac imaging are rapidly improving the reproducibility of this approach and the possibility to reduce time necessary to generate a report.

In cardiac computed tomography angiography (CCTA) the main application of AI in clinical practice is focused on detection of stenosis, characterization of coronary plaques, and detection of myocardial ischemia.

In cardiac magnetic resonance (CMR) the application of AI is focused on post-processing and particularly on the segmentation of cardiac chambers during late gadolinium enhancement. In echocardiography, the application of AI is focused on segmentation of cardiac chambers and is helpful for valvular function and wall motion abnormalities.

The common thread represented by all of these techniques aims to shorten the time of interpretation without loss of information compared to the standard approach.

In this review we provide an overview of AI applications in multimodality cardiac imaging.

## 1 Introduction

Cardiovascular imaging represents a wide field of application in diagnostics in medicine characterized by a fast technological improvement in order to satisfy the pressing clinical and therapeutical needs [1, 2, 3, 4, 5, 6, 7]. Despite this strong push and the attempt to perform comprehensive evaluations, the clinical and prognostic value of current traditional imaging tools is limited owing to different reasons including intra and interobserver variability, suboptimal image quality, time-consuming exams, operators fatigue and so on [8, 9, 10]. On parallel, the application of artificial intelligence (AI) in medical imaging has been rapidly growing during recent years [11, 12], especially with developing of Machine learning (ML) and deep learning algorithms [13]. ML needs that some features are provided manually by the user while in deep learning (DL) the same features are automatically extrapolated by the algorithm [13].

Focusing on cardiovascular imaging, the application of AI in the field ranges from image acquisition, to image analysis, and then ultimately to evaluation and prognosis [13, 14, 15, 16, 17]. The main advantage of AI applications in cardiovascular imaging is the possibility to provide optimal image quality, fast image analysis, and prognostic stratification in a relatively brief time, with high reproducibility, and with low involvement by the reader [13, 14, 16].

In cardiac computed tomography (CT) the application of AI can be useful in both non-contrast and contrast-enhanced acquisitions [13].

Beyond the role of AI in the evaluation of calcium scores [18, 19], the main application of AI in cardiac CT imaging is the analysis of cardiac computed tomography angiography (CCTA) to evaluate the severity of coronary stenosis [15], evaluation of coronary plaques [20] and allowing thorough evaluation of myocardial ischemia with Fractional Flow Reserve CT (FFR-CT) and myocardial perfusion (MP) [21, 22, 23, 24, 25].

In Cardiovascular Magnetic Resonance (CMR) the application of AI is well established to be useful in image acquisition [26, 27]], as well as post-processing in both ventricular function [28] and tissue characterization [14].

In echocardiography, the application of AI can be useful for the automatic evaluation of biventricular volumes, function, strain analysis, wall motion, and power doppler acquisition [29].

AI in cardiac imaging and analysis has a potential role in terms of prognostic stratification, especially if imaging findings are combined with clinical data [16].

The aim of this review is to show the application of AI in multimodality imaging represented by cardiac CT, CMR, and echocardiography. All patients signed informed written consent for this review.

## 2 AI algorithms for cardiovascular imaging

Deep learning is a class of artificial intelligence methods that can learn abstract representations from structured and unstructured data, such as images [30]and bio-signals [31]. Deep learning models are a boosted version of the first generations of neural networks in which specialized layers of neurons, able to handle any source of data, have been embedded. In the imaging field, the ‘convolutional layer’ plays a central and crucial role centered on its ability to identify relevant patterns in images, such as edges, color gradients, and shapes, by performing the convolution operation between image and a set of specific filters. The pattern learned by the network is subsequently used to face two kinds of analysis: classification or segmentation. Classification aims to discriminate between two or more classes of patients, while segmentation aims to detect specific structures or objects by performing pixel-level labeling.

Corresponding to the task to be accomplished, specific neural network architectures have been proposed. Convolutional Neural Networks (CNN) and Recurrent Neural Networks (RNN), such as the Long-Short Term memory (LSTM) and the Gated Recurrent Units (GRU), are commonly implemented to perform classification tasks. Whereas, Unet, Generative-Adversial Networks (GANs) and auto-encoders (AE) are widely used for segmentation analysis.

For instance, Betancur et al. [32] developed a standard CNN network with 3 convolutional layers to produce reproducible tomography of myocardial perfusion imaging in more than 2 thousand patients to predict major adverse cardiac events (MACE) after 3 years follow-up. This simple architecture was found to be superior to existing methods for predicting outcomes. Moreover, Tison et al. [33]trained more complex CNN architecture with 8 consecutive convolutional layers on about 10 thousand 12-lead ECGs. The network showed an AUC = 0.97 vs. 0.91 of current ECG algorithms. Choi et al. [34]employed a GRU neural network to detect new onset heart failure (HF) from electronic health records combined with temporal information. These networks were shown to have c-statistic for incident of HF of 0.78 at 12-month observation and 0.88 at 18-month observation.

Using the segmentation method, Zreik et al. [25] applied a combined strategy consisting of an autoencoder and a support vector machine to automatically detect coronary stenosis in rest coronary 10.13039/100004811CT angiograms, which showed great improvement compared to fractional flow reserve (10.13039/501100006148FFR) measurements. Bai et al. adapted a VGG-16 network to segment left and right ventricles on 100,000 MRI images from the UK Biobank [35]; and Avendi et al. [36] developed a stacked autoencoder to infer the LV shape obtaining a relevant improvement over the existing manual methods.

Regarding informatic resources, few effective frameworks have been developed to build end-to-end deep learning models. At this time, the three most used frameworks are Tensorflow, Keras, and PyToch [37]. Tensorflow, developed by Google's Brain team, supports languages like Python and R, and uses dataflow graphs strategically to effectively process data. Keras is an R/Python package which provides high-level functions to easily build Tensorflow or Theano models as a stack of consecutive layers. Finally, PyTorch developed by Facebook's AI Research lab employs Python along with CUDA and was designed to scale both the production of building models and overall flexibility.

## 3 AI in cardiac computed tomography

The application of AI in cardiac CT ranges from diagnosis to prognosis [13, 38] and seems likely to play a key role in the future for speeding up the time of reporting [15, 39], providing information regarding coronary plaques [20], and detection of myocardial ischemia [21, 40].

### 3.1 Calcium score

The coronary calcium score (CAC) is one of the best predictors of coronary artery disease (CAD) outcome and a validated tool for prognostic stratification [41, 42, 43]. Currently, the calcium score is acquired using ECG-gated non-contrast acquisitions [44] and subsequently, images are commonly analyzed using semi-automated software, which requires time [13]. A fully automated approach would be extremely helpful in clinical practice, however until now these approaches have been inaccurate and poorly reproducible [18].

The application of AI in the evaluation of CAC would be extremely helpful in clinical practice because it would provide clinicians information regarding the prognostic stratification and probability of significant CAD in a relatively abbreviated time [16].

One of the first approaches described in the literature regarding the application of AI in the evaluation of CAC was performed by Isgum et al. [45]; the authors analyzed a pool of images obtained from ECG gated non-contrast images and they correctly identified the patient's risk score group in 93.4% of cases and identified coronary calcification in 73.8% of cases [45].

Subsequently, Sandsted et al. compared the semi-automated approach with a fully AI automated technique for the evaluation of CAC [46]. Interestingly, the authors found a Spearman's rank correlation coefficient between AI and semiautomated software for Agatston Score, Calcium Volume score, and Calcium Mass of 0.935, 0.932, and 0.934 respectively, while intraclass correlation were of 0.996, 0.996, and 0.991 respectively [46].

Recently a manuscript has been published by Winkel et al. showing that the application of deep learning (DL) software in a multicenter study which was able to obtain values of CAC compared with human readers in a vessel analysis with an accuracy of 93% and in the absence of calcium was found to have a sensitivity, specificity, and accuracy of 97%, 93% and 95% respectively [47].

The application of AI in the evaluation of CAC is primarily focused on the evaluation of ECG-gated non-contrast images, yet there are several manuscripts emerging in the literature that are exploring the possibility to calculate calcium scores from chest CT scans without any ECG-gated acquisition [19, 48, 49].

Takx et al. recently analyzed the impact of CAC analysis in patients who underwent non-contrast CT acquisition for the evaluation of lung cancer screening [50]. In a cohort of 1793 patients, the authors found a good reliability with a weighted *k* of 0.85 for the Agatston risk score between manual and automated software [50]; however, the automated approach showed an underestimation of calcium volume if compared with manual software [50].

These analyses exemplify the key role of AI for the evaluation of CAC which will be fundamentally focused on the evaluation of images acquired without ECG-gating allowing an evaluation of CAC score with high reproducibility.

### 3.2 Coronary stenosis

It has been demonstrated that CAD-RADS is an excellent tool for the classification of stenosis using a model where patients undergoing CCTA are able to be risk stratified given their specific CAD-RADS score [51]. The evaluation of CAD-RADS has an important impact in terms of prognosis [52] with a 5 year event-free survival of 95.2% in patients with a CAD-RADS score of 0 compared to 69.3% of patients with a CAD-RADS score of 5 [52].

The possibility to provide the CAD-RADS score in a quick and automatic way would represent a fundamental tool during the reporting of CCTA [15]. Considering the expected increase in CCTA examinations during the years ahead, it would be important to at least have an algorithm that can adequately differentiate patients with CAD-RADS = 0 and CAD-RADS ≠ 0 [15].

One of the first applications of CAD-RADS classification using a deep learning algorithm was described by Muscogiuri et. al [15]. The authors developed three models: Model A (CAD-RADS 0 vs CAD-RADS 1-2 vs CAD-RADS 3,4,5), Model 1 (CAD-RADS 0 vs CAD-RADS>0), Model 2 (CAD-RADS 0-2 vs CAD-RADS 3-5) [15]. The sensitivity, specificity, negative predictive value, positive predictive value and accuracy for Model A, Model 1 and Model 2 were respectively: 47%, 74%, 77%, 46% and 60% (Model A); 66%, 91%, 92%, 63%, 86% (Model 1); 82%, 58%, 74%, 69%, 71% (Model 2) [15]. Furthermore, it is not surprising that the algorithm provided a CAD-RADS score in a significantly shorter time compared to human readers (104.3 ± 1.4 s vs 530.5 ± 179.1 s, p = 0.01). The most important findings demonstrated by the work of Muscogiuri et al. is the high diagnostic accuracy of the algorithm for the differentiation between patients with CAD-RADS >0; this finding can be extremely important in clinical routine helping to speed up the reporting of CCTAs.

Another article regarding coronary stenosis evaluation was written by Paul et al. [53].

The authors developed a model that was able to predict stenosis <50 and ≥50% based on deep learning, CAD-RADS classification, and MPR reconstruction [53] which reached a sensitivity, specificity, positive predictive value, negative predictive value and accuracy, respectively, of 93%, 97%, 93%, 97% and 96% in a patient-based model [53].

Also, Xu et al. developed a deep learning model centered on the evaluation of coronary stenosis as compared to ICA [54]. The sensitivity, specificity, positive predictive value and negative predictive value that the authors observed for deep learning algorithm vs readers for detection of stenosis >50% were, respectively, 58.6%, 92.2%, 83.6%, and 76.7% via vessel-based analysis, while they were 84.0 %, 71.0 %, 93.6% and 46.7% via patient-based analysis [54]. Furthermore, the authors showed a significant reduction in time of analysis if compared to manual work (p < 0.01) [54].

Like the results of the previous manuscripts mentioned above, Choi et al. developed an algorithm that was able to identify stenosis >70% with an accuracy, sensitivity, specificity, positive predictive value and negative predictive value of 99.7%, 90.9%, 99.8%, 93.3%, 99.9% respectively [55]. Meanwhile, Griffin et al. tested an AI-algorithm that was able to identify stenosis with a sensitivity, specificity, positive predictive value, negative predictive value, and accuracy of 94%, 82%, 69%, 97%, and 86% respectively for stenosis ≥70% [56].

Analysis of stenosis using AI, regardless of CAD-RADS scores, has clear potential as an interesting field with suspected high clinical impact in the future. The application of a deep learning algorithm in CCTA reporting will undoubtedly be helpful for reduction of reporting time, particularly by avoiding the examinations of CAD-RADS scores of 0.

**Figure 1:**
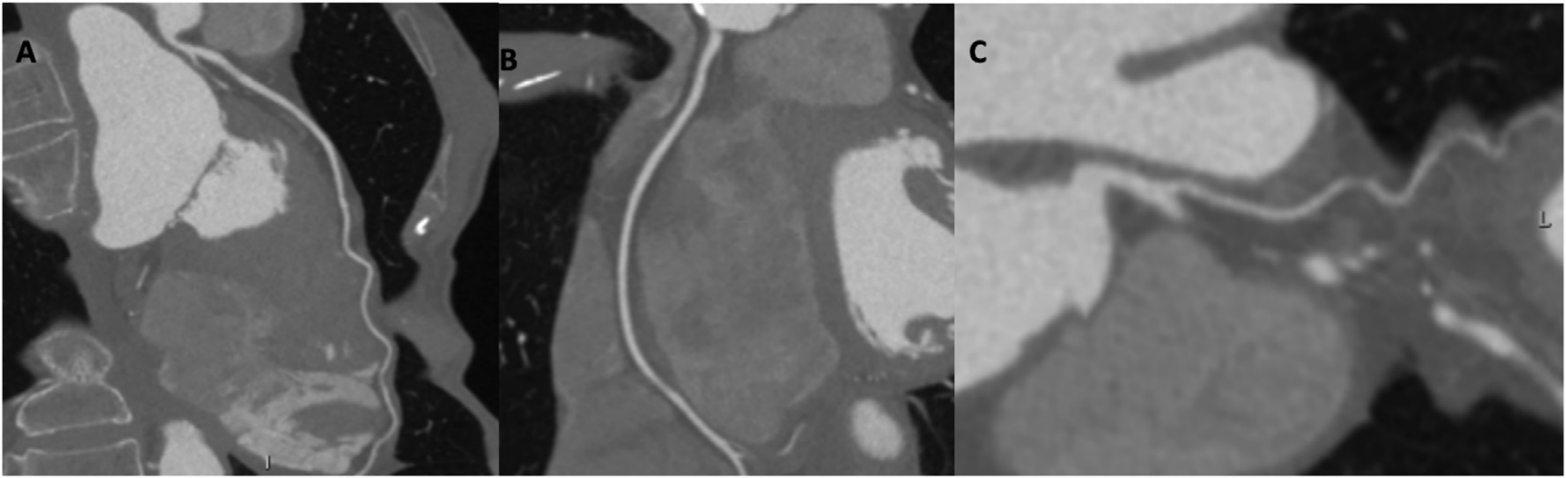
64-year-old female patient underwent to coronary computed tomography angiography for dispnea. The left main and left anterior descending artery showed no atherosclerosis (A) as well as right coronary artery (B) and circumflex (C).

**Figure 2:**
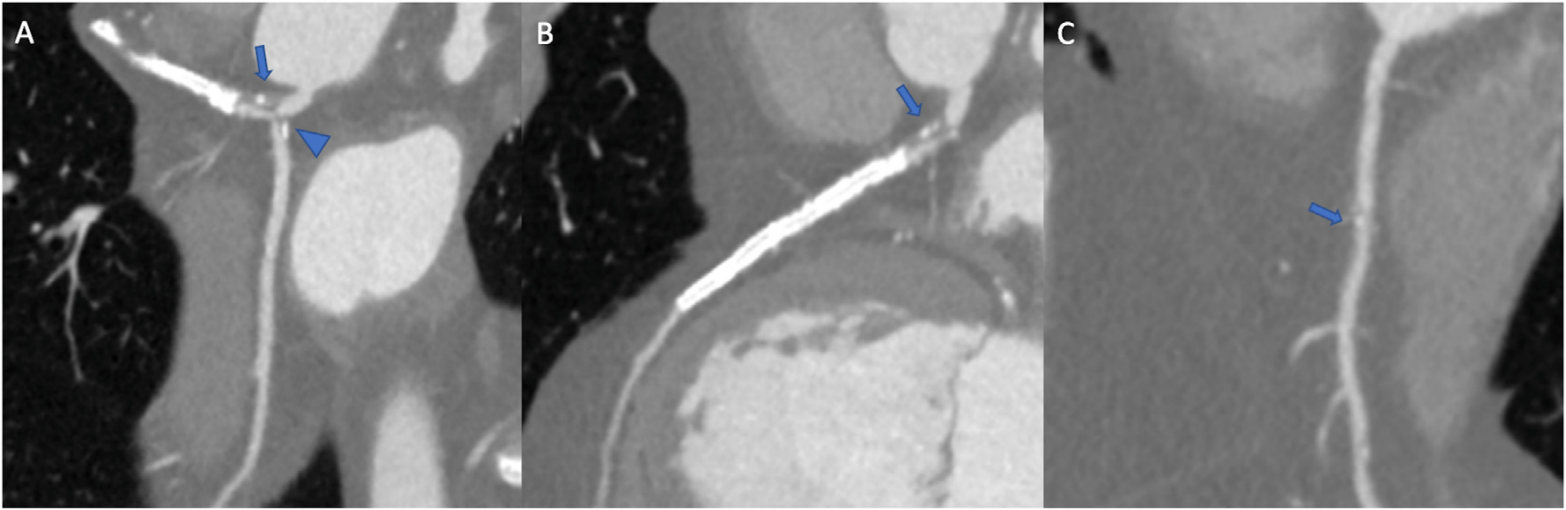
62-year-old male patient underwent coronary computed tomography angiography for chest pain in patient with previous PTCA and stent on left anterior descending artery. Left main coronary artery (A, arrow) and circumflex (A,arrowhead) show severe ostial mixed plaque, as well as left anterior descendant (B, arrow); the right artery only shows moderate calcific plaque at mid segment (C, arrow).

A case of CAD-RADS 0 and has been shown in Figure 1 (A-C) and a case of CADRADS 5 has shown in Figure 2 (A-C).

### 3.3 Plaque analysis

In cardiac computed tomography, AI may have several strengths in coronary atherosclerotic plaque analysis including the promise of providing more efficient and rapid methods for characterization of plaque morphology [13, 16, 20].

As described above, AI may be useful in helping with coronary calcium score evaluation (CACS). In parallel with this assessment, AI may simplify the assessment of atherosclerotic plaque vulnerability using its characteristic features. Choi et al. proposed an AI algorithm to evaluate vessel morphology and degree of stenosis, comparing the new model with the consensus of three expert readers [55]. The authors reported that the AI model achieved an excellent performance in detecting a degree of stenosis >70% with accuracy, sensitivity, specificity, positive predictive value and negative predictive values of 99.7%, 90.9%, 99.8%, 93.3%, and 99.9%, respectively with excellent agreement between expert readers and AI (intraclass correlation coefficient = 0.91) [55]. In addition, the authors demonstrated that AI detected and quantified high-risk plaque features more often than that of expert readers (21,1% vs 13,4 %, respectively) [55]. Similarly, Masuda et al used a ML algorithm with a histogram analysis for the identification of fibrous, fatty, or fibrous-fatty plaques showing an accuracy of 0.92 in comparison with an accuracy of 0.83 of the conventional CT method [57].

In addition, AI algorithms have been proposed for the assessment of ischemia risk scores based on computed tomography angiography imaging. In particular Dey et al. investigated a ML approach with quantitative plaque metrics using CTA to measure the functional significance of coronary stenoses in comparison with FFR ^7^. The authors reported that an integrated ML algorithm combining quantitative CTA measures showed a higher area under the curve (0.84) than individual quantitative CTA metrics, stenosis (0.76), low-density non calcified plaque (0.77), and total plaque volume (0.74) [58]. The authors concluded that a combined ischemia risk score based on an integrated ML approach from combining quantitative CTA measures improved the prediction of lesion-specific ischemia ^7^.

Finally, the application of AI can be extended beyond plaque quantification to include adjacent tissues. Commandeur et al. evaluated the performance of DL models for fully automated quantification of epicardial adipose tissue from cardiac CT. The proposed models achieved a quantification in a mean time of 1.57 s compared to 15 min for human observers with high agreement between the automated method and the observer (R = 0,905, p < 0001) [59].

**Figure 3:**
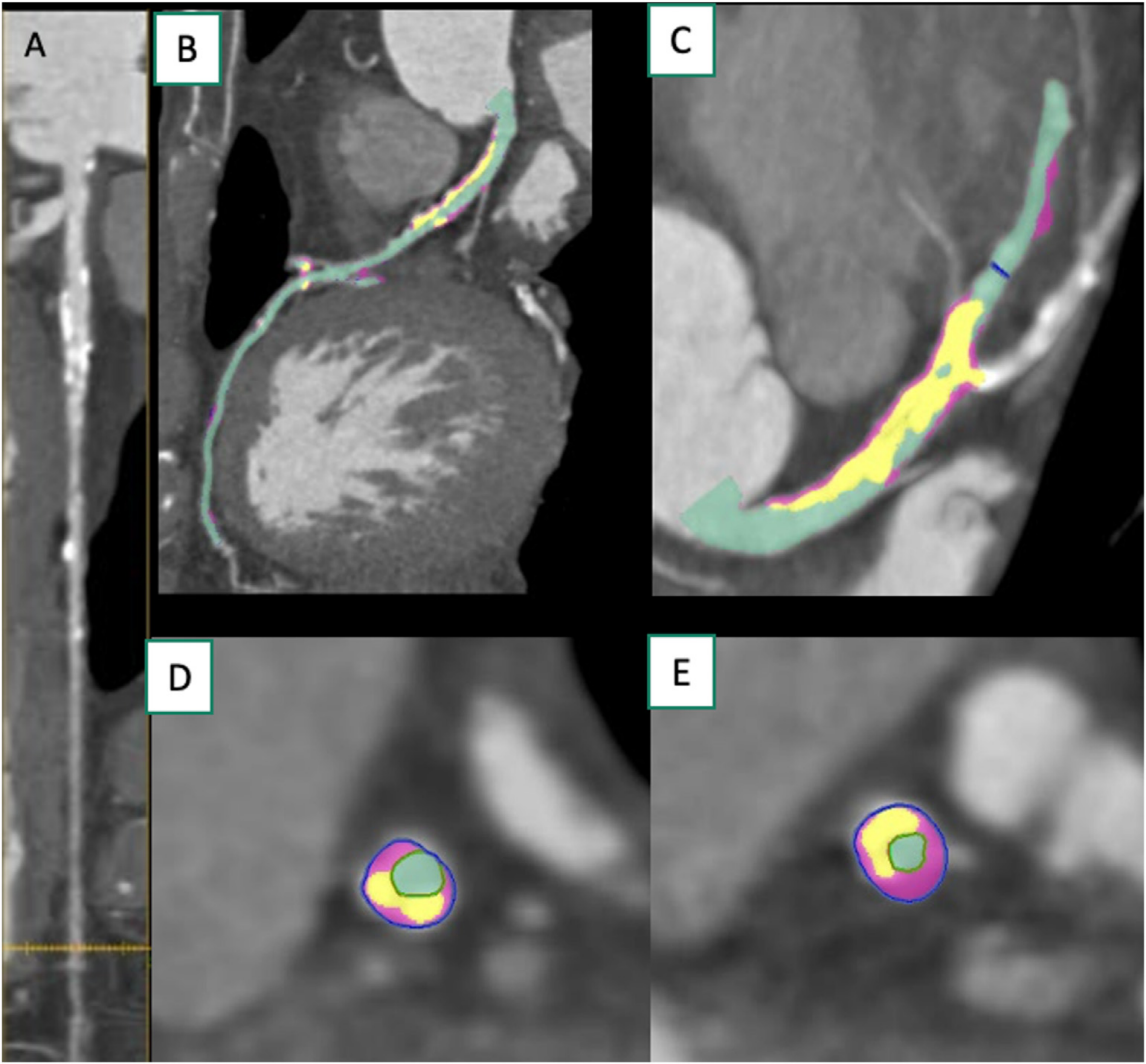
An example of quantitative plaque AI -based measurements in a 58-year-old make with exertional chest pain. A straightened MPR demonstrated a diffuse calcified atherosclerotic plaque in left circumflex artery (A). The curved multiplanar reformatted image of the left anterior descending artery, with different plaque components (B, C). The cross-sectional view of the proximal left anterior descending artery (D) and of the mid left anterior descending artery (E) demonstrated different plaque components.

A case has been shown in Figure 3 (A-E).

### 3.4 FFRct and myocardial perfusion

Fractional flow reserve Computed Tomography (FFRct) is a useful tool that increases the positive predictive value of CCTA [60, 61]. FFRct using a fluid-dynamic model evaluates the potential for ischemia caused by coronary plaques [62]. In recent developments, the application of machine learning algorithms for the evaluation of FFRct has been extremely helpful for the evaluation of different ischemic lesions [22, 57, 63, 64].

Clinical validation of ML-FFRct has been published in the literature by the MACHINE Registry [65].

In a multicenter registry across the United States, Europe, and Asia, the accuracy of ML-FFRct has been compared with invasive FFR as a reference standard [65]; furthermore, the results of ML-FFRct were compared also with values of FFRct obtained by the classic fluid-dynamic FFRct (CFD-FFRct). A comparison of ML-FFRct and CFD-FFRct showed the same area under the curve (AUC: 0.84) compared to the low AUC of CCTA (AUC: 0.69) [65]. On a vessel-based analysis the diagnostic accuracy of ML-FFRct reached 78% compared to 58% of CCTA while the patient accuracy reached 85% compared to 71% of CCTA [65]. The results of this multicenter study were confirmed by a single-center study [21, 23] that demonstrated the increased diagnostic accuracy of ML-FFRct compared to CCTA alone.

The advantage of ML-FFRct for diagnostic accuracy has been shown compared to CCTA in the presence of calcified lesions [66]. With an Agatston score ≥400, the AUC increased from 0.55 of CCTA to 0.71 for ML-FFRct. With an Agatston score between 0 and 400, the AUC increased from 0.63 of CCTA to 0.86 of ML-FFRct [66]. Interestingly, the ML-FFRct showed also an increased prognostic value in short follow up (1 year) for development of MACE if compared to CCTA (p < 0.04).

Like FFRct, myocardial CT perfusion (CTP) can be extremely useful for detection of ischemic coronary plaques [67, 68, 69]. The application of AI in CTP is still very limited, though there are some interesting articles recently published in the literature [24, 40]. Xiong et al first described this application using an ML algorithm trained on normalized perfusion intensity, transmural perfusion ratio, and myocardial wall thickness. It demonstrated a better performance with AdaBoost producing a sensitivity of 0.79 and specificity of 0.64 if compared to quantitative coronary angiography [24]. Another interesting article on the application of ML in CTP, was published by Han et al. [70]. The authors developed an ML algorithm that was able to identify deficits of perfusion at rest using datasets from CCTA with an accuracy, sensitivity, specificity, positive predictive, and negative predictive of 68.3%, 52.7%, 84.6%, 78.2%, and 63.0% respectively in patient-based analysis [70].

Recently Muscogiuri et al. developed a DL algorithm that was able to identify ischemic myocardium at rest on CCTA and in addition to anatomical evaluation with a sensitivity, specificity, NPV, PPV, accuracy, and AUC respectively of 100%, 72%, 100%, 74%, 84%, 96% [40]. Though the populations these methods were applied to were small, this article highlights the possibilities available to develop algorithms that provide quality information about the presence of ischemic myocardium using the appropriate Hounsfield unit for ischemic myocardium at rest.

**Figure 4:**
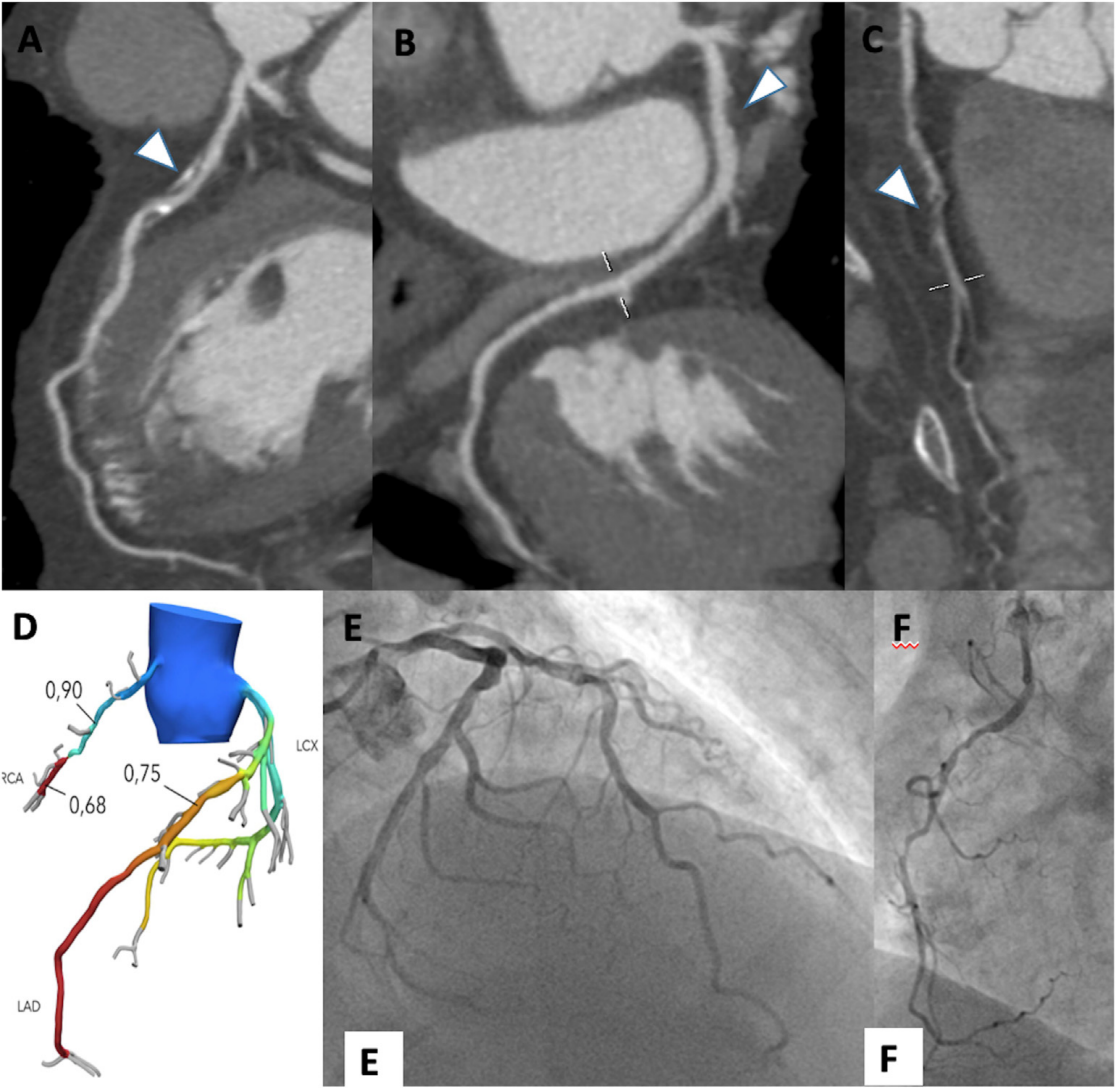
70-year-old male patient underwent to coronary computed tomography for chest pain. Left anterior descending artery shows a severe mixed plaque stenosis (A, arrowhead); left circumflex artery shows a moderate proximal fibro-lipid plaque (B, arrowhead) while right coronary artery shows a severe fibro-fatty plaque stenosis (C, arrowhead). The FFR_ct_ assessment confirmed the functional significance of the stenosis on left anterior descending artery and right coronary artery (D), while FFR_ct_ values of the left circumflex artery were above the ischemia threshold of 0.80. The invasive coronary angiogram shows severe stenosis of the mid segment of left anterior descending artery (E) and mid segment of right coronary artery.

**Figure 5:**
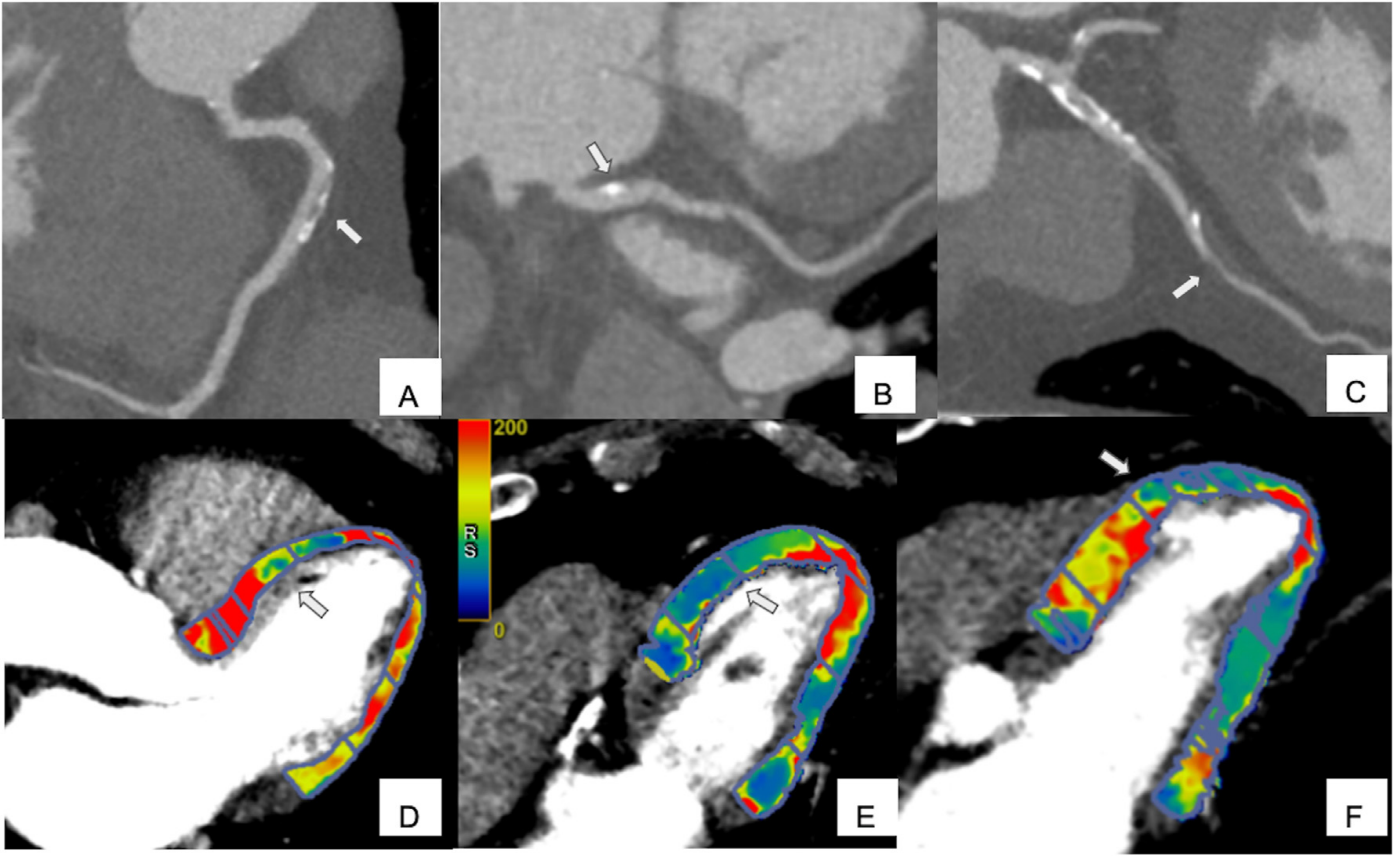
81-year-old male patient underwent perfusion CT for atypical pain and dispnea. Right coronary artery (A, arrow) and left circumflex (B, arrow) shows mild mixed plaque while left anterior descending artery demonstrates severe fibrofatty stenosis (C, arrow). The findings are then confirmed by the perfusion study, which shows antero septal mid-ventricle (D, arrow) and anterior mid-ventricle (E, arrow) and anterior apical segment (F, arrow).

Application of FFRct and CTP have shown respectively in Figure 4 (A-F) and 5 (A-F)..

## 4 AI in cardiac magnetic resonance

The application of AI in CMR ranges from image acquisition to image analysis [13, 27].

The main application of AI in post-processing has been focused on segmentation and tissue characterization [13, 71].

### 4.1 Function

Evaluation of cardiac function is extremely important in CMR considering the impact related to prognosis [1, 72]. Several techniques have been described evaluating biventricular cardiac volumes and function from cine images, however all these manual or semi-automated approaches require time-consuming analysis [35, 73]. The application of fully automated software based on AI should decrease the variability of volumes between readers and concurrently increase the speed of reporting [13].

Several approaches have been shown in the literature demonstrating differences in accuracy when evaluating the volumes resulting in competition and comparison [74].

Isensee et al. took part in the competition at the 20th International Conference on Medical Image Computing and Computer Assisted Intervention (MICCAI) and showed that the algorithm they developed based on 2D and three-dimensional (3D) U-net model had the highest Dice coefficients in the diastolic phase for the left ventricle (0.96), right ventricle (0.94), and myocardium (0.90) [75]. Several research groups have developed algorithms for cardiac segmentation based on the population of the MICCAI competition [74]; however, these approaches have been limited due to a small population [74]. In order to overcome this limitation, Bai et al. proposed an algorithm of analysis based on a larger population coming from the UK biobank [35]. The authors developed an algorithm consisting of a fully convolutional neural network (FCN) trained on cine images which revealed high DICE coefficients for the left ventricle cavity (0.94), left ventricular myocardium (0.88), and right ventricle (0.90) [35]; furthermore, the authors demonstrated a close correlation between biventricular volumes and left ventricular mass compared to manual segmentation [35].

Interestingly, Penso et al. using a large dataset, developed a U-Net that was able to segment images in a cohort of patients with three different cardiac phenotypes with good agreement compared to manual segmentation resulting in a DICE score for left ventricle, right ventricle and myocardial mass respectively of 0.94, 0.90 and 0.85 [28].

The main characteristic of automatic segmentation is represented by the possibility to obtain results like manual segmentation in a short time; therefore, it is not surprising that several software vendors are pushing this AI solution for the analysis of volume and function.

**Figure 6:**
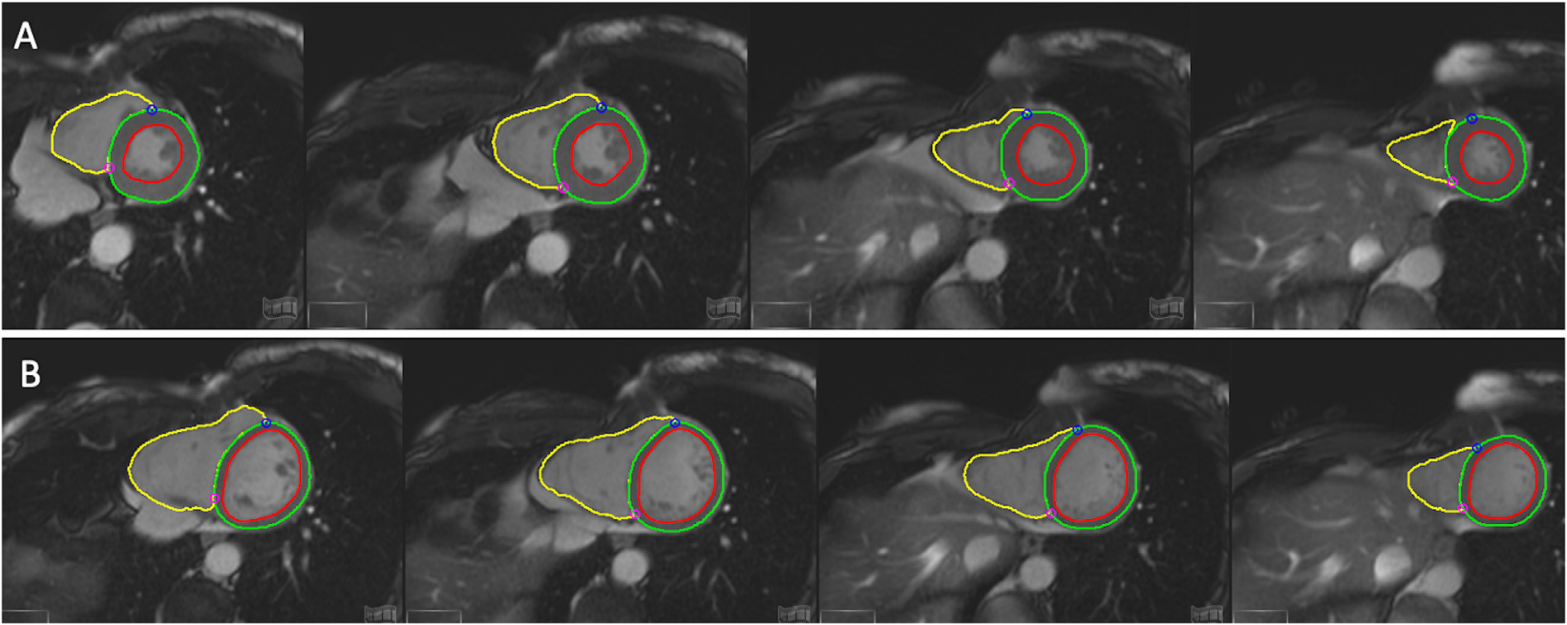
19-year-old male patient underwent cardiac magnetic resonance for follow-up of a COVID19 related myocarditis. Deep learning algorithm provided contours of left endocardium (red line), epicardium (green line), and endocardium of right ventricle (yellow line) in systolic phase (A). The same contours were automatically depicted on systole (B).

A representative case is shown in Figure 6 (A-B).

### 4.2 Tissue characterization

One of the main advantages of CMR over the other techniques are the ability to deeply evaluate and characterize tissues [76, 77, 78]. T1-weighted black blood sequences are mainly focused on the evaluation of fibro-fatty infiltration and cardiac masses [79, 80], while T2-weighted images are mainly focused on the evaluation of myocardial edema [80, 81].

T1-mapping with ECV sequences can provide information regarding the extracellular volume [77, 82] while T2-mapping can provide the presence of myocardial edema [83, 84]. Another method that is useful for the evaluation of myocardial fibrosis and decreased gadolinium wash-out is the acquisition of late gadolinium enhancement (LGE) sequences [13]. These sequences are based on different settings of cardiac disease so using LGE can be helpful in terms of prognostic stratification and planning of therapy [76, 85, 86, 87].

LGE can be quantified using manual contouring or semi-automated software however, like the evaluation of volumes, this approach is frequently time consuming [13].

In order to overcome this issue, an automatic approach was developed by Zabihollahy et al.The authors validated a 3D-CNN segmentation using a 3D LGE dataset of the left ventricle [88]. Comparing the data of 3D-CCN with manual segmentation showed a Dice similarity coefficient (DSC) of 0.94 [88]. Moccia et al. developed a segmentation model based on a fully convolutional neural network for LGE segmentation and provided a sensitivity, specificity, accuracy, and DSC of 88.1%, 97.9%, 96.8%, and 71.3%, respectively. Another interesting approach was shown by Zhang et al. [89]the authors developed an algorithm that was able to identify LGE from cine images showing a sensitivity, specificity, and AUC of 89.8%, 99.1%, and 0.94% respectively on non-contrast cine images [89]. As highlighted in the manuscript of Zhang et al [89]the possibility to obtain images of fibrosis from cine images represents an interesting tool considering the future application of AI in non-contrast images.

Another interesting approach using deep learning was developed for atrial segmentation of scar by Li et al. [90]. The authors found that their network provided an accuracy of 0.86% ± 0.03% and a mean DSC of 0.70 ± 0.07 [90].

Automatic segmentation of myocardial and atrial LGE represents an important tool for the reporting of CMR providing medical teams the opportunity to obtain key information regarding prognosis in a short time.

## 5 Artificial intelligence in echocardiography

Due to its worldwide availability, echocardiography is usually the first-level imaging technique in the setting of cardiovascular disease [91]. Accurate and reproducible measurements are of crucial importance in the diagnostic work-up and during the follow-up of several cardiac disorders and might significantly impact clinical decision-making [92, 93]. Despite advancements in echocardiography in recent years, and the development of three-dimensional (3D) software packages for strain analysis [94, 95], the operator dependency of acquisition and interpretation of imaging data remains a major drawback [96]. The assessment of left ventricular ejection fraction (LVEF), one of the most important echo parameters on which many clinical decisions rely [97], has been shown to be closely influenced by the reader's experience, commonly resulting in a subjective interpretation [98]. Using AI might create new clinical advantages - shifting image interpretation from a subjective to an objective field, thereby resulting in more accurate and reproducible analyses.

Additionally, the development of new technologies has led to an increasing number of parameters that can be derived during each echocardiographic examination, but this growing degree of complexity might be difficult to understand by the ordering physician. Arterial hypertension is one of the most common cardiovascular diseases requiring echocardiographic evaluation [99]. In these patients, the assessment of myocardial mass, volume, and function is of particular importance. On top of the standard parameters, measurement of left atrial (LA) strain might detect early diastolic dysfunction and LV strain could uncover early systolic dysfunction. The LV strain pattern may help in the differential diagnosis between hypertension and infiltrative disease requiring a second-level imaging study [100, 101]. In day-to-day practice, it can be difficult for the clinician to manage the multitude of information provided and correctly link the data to each other. AI has potential to improve the analysis of an exponential amount of information at an advanced level of interpretation, helping to improve patient diagnosis and prognostic stratification.

Finally, it is noteworthy that the high workflow in many of the echo labs around the world likely increases the risk of medical errors that could be avoided, or at least reduced through using a double-checking surveillance system potentially provided using AI.

However, despite achievable advantages in image analysis and interpretation, AI may also guarantee wider access to care and help reduce the cost of undergoing testing via a primary imaging modality such as echocardiography [102].

**Figure 7:**
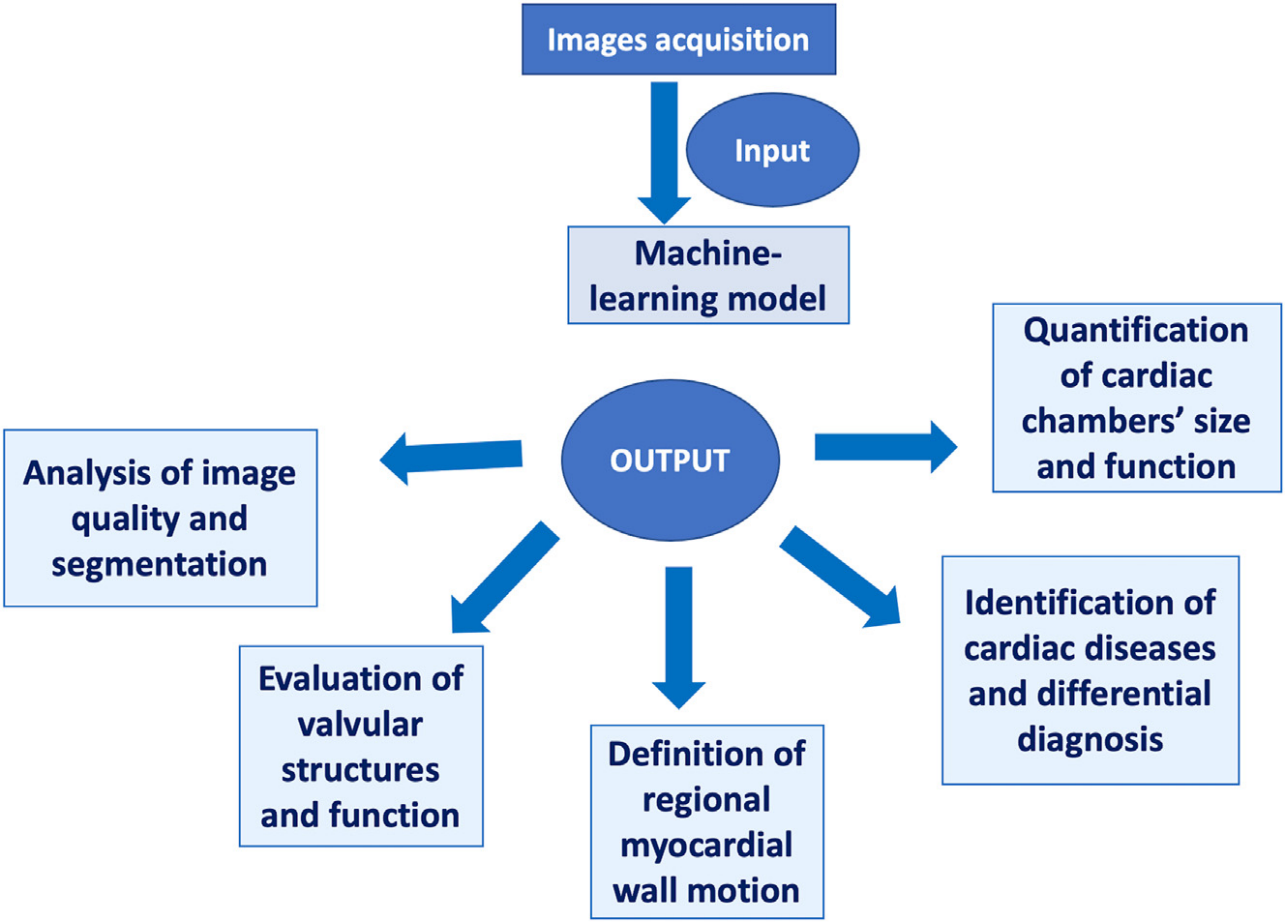
After the acquisition of images by the operator, Artificial Intelligence is able to provide a wide spectrum of critical information ranging from the simple definition of image quality and segmentation of cardiac structures to more complex processes such as the evaluation of valvular diseases or differential diagnosis between cardiovascular diseases. This may support the daily practice in echo lab, improving diagnostic accuracy and reproducibility with a reduction of the examination time.

A representative figure for the application of AI in echocardiography was shown in Figure 7.

### 5.1 AI in image localization and segmentation

Appropriate automated detection of adequate echocardiographic views featuring the cardiac chambers are the primary focus of automated echocardiographic analysis, and several papers have already shown the use of AI to allow accurate view identification. In a recent study by Zhang et al, the authors used a deep learning (DL) model with a convolutional neural network (CNN) to derive a view classification system and image segmentation model derived from more than 270 echocardiograms and more than 700 images. It was subsequently deployed on more than 14,000 echocardiograms where the authors found that AI correctly identified 23 viewpoints, including those of suboptimal image quality, and correctly performed chamber segmentation using the 5 most common views with an accuracy up to 96% [103]. Similarly, DL with CNN was used in another recently published study in which a view classification model trained on over 800,000 images showed an accuracy up to 97.8% for 15-view classification [104].

Based on these results, the application of AI in view and segmentation echocardiographic models seem to be promising, with differences in vendors and poor image quality representing the major limitation in reaching perfect accuracy [105].

### 5.2 AI in analysis of cardiac chambers’ size and function

Analysis of cardiac chambers’ size and function is the primary goal in the current practice of echocardiography. Accurate measurement of cardiac volume, mass, and function requires trained readers. Accuracy is known to be affected by a high degree of interobserver variability and likely affected by errors in a high workflow lab [98]. In direct comparison between automated and manual measurement of LV wall thickness, LV and LA volume, and global longitudinal strain (GLS), more than 8,000 two-dimensional (2D) echocardiographic images have shown a median absolute deviation between 15 and 17% for mass and volumes, while LVEF and GLS showed smaller differences with a median absolute deviation of 6% and 1.4%, respectively [103].

However, since 3D echocardiography has proved to provide more accurate and reproducible results compared to 2D, there has been a push for the development of fully automated 3D software packages that allow direct measurement of cardiac mass, volume, and function without reader influence. There is currently several commercially available software packages available for 3D analysis of the left ventricle (LV), left atrium (LA), and right ventricle (RV), primarily based on adaptative analytics algorithms [106] or probabilistic contouring algorithms [107]. Several studies have demonstrated how the use of these automated software packages provide accurate analysis of cardiac chamber volume, and function, as well asLV mass similar to cardiac magnetic resonance imaging [106, 108, 109, 110, 111, 112]. Particularly, the study by Narang et al., demonstrated how the use of fully automated software packages for the assessment of LV/LA volume and function results also in time-saving analyses with a mean time of 35 ± 17 s for automated analysis compared to 3.6 ± 0.9 min and 96 ± 14 min for semi-automated 3D echo and CMR analysis, respectively [106].

As for view and segmentation models, the use of AI for automated assessment of cardiac chamber's size and function is still limited by the quality and quantity of the image datasets used in the training process. This has been reflected by suboptimal analysis of volume and function in patients with distorted LV shape secondary to the small number of those pathological datasets available for machine learning training [105].

### 5.3 AI in assessment of valvular function

Of particular importance in the setting of echocardiography is the Doppler-analyses using pulsed wave and continuous wave Doppler settings. Inaccuracies in assessment of Doppler velocity and tracing may lead to overestimation or underestimation of valvular disease. In a study by Gaillard et al., an automated detection of wave contour velocity based on active contour models measured at the aortic valve and LV outflow tract which showed good agreement compared to manual tracing in both patients with sinus rhythm and atrial fibrillation [113]. Recently, the use of unsupervised models for automated detection of bioprosthetic aortic valve degeneration has been shown to have a high sensitivity for the detection of valve degeneration which can be particularly useful in the follow-up of patients with aortic valve replacement [114]. Additionally, the use of AI has been shown to be promising in the appropriate selection of patients eligible for mitral valve repair [115].

### 5.4 AI in assessment of myocardial wall motion

Assessment of regional wall motion abnormalities (RWMA) is one of the central clinical concerns that need to be investigated when using echocardiography for the diagnosis and management of coronary artery disease. However, as already demonstrated, assessment of myocardial motion is subjective and heavily dependent on the operator's experience [116]. A recent study by Kusunose et al. showed how the use of DL algorithms, specifically a CNN model, was able to identify RWMA with an accuracy similar to that of experienced sonographers (AUC 0.99 for DL model vs 0.98 for sonographers), but the accuracy of the algorithm was significantly higher when compared to RWMA detection by fellowship-trained interpreters (0.99 vs 0.90, respectively) [105]. There was a lower yet still highly accurate detection of RWMA shown using a CNN model in patients undergoing 3D echo Dobutamine stress tests compared to experienced readers [117].

### 5.5 AI in assessment of cardiac disease

The primary aim when performing echocardiography is the early detection of several disease statuses in patients with cardiovascular risk factors or cardiovascular morbidities. However, frequently to achieve this goal several echocardiographic studies are required. In this setting, the use of AI may facilitate prompt diagnosis with low-cost workflows. Currently, several AI-based models for the early detection of cardiac dysfunction or disease identification have been trained. ML based algorithms using echocardiographic parameters at rest and during exercise have been demonstrated to improve the accuracy in the diagnosis of heart failure with preserved ejection fraction [118].

In a recent paper by Zhang et al., the authors trained a CNN black-box models to detect hypertrophic cardiomyopathy, amyloidosis, and pulmonary arterial hypertension. The resulting models showed high diagnostic accuracy in disease diagnosis (AUC 0.93, 0.87, 0.85, respectively) [103]. Sengupta et al. demonstrated how the use of a cognitive machine learning (ML) model based on clinical and multimodality imaging data allows proper differentiation between constrictive pericarditis and restrictive cardiomyopathies with an accuracy of 96% [119]. The use of ML model is also promising in differentiation between athlete's heart and hypertrophic cardiomyopathy [120].

### 5.6 Future perspective

The application of AI in cardiovascular imaging as described above is rapidly increasing considering the possibilities to reduce reporting times while doing so with high accuracy. In this review, the potential applications of AI algorithm have been described focusing on the impact that these algorithms could have in clinical practice. These algorithms can improve the diagnostic accuracy leading to a model of “precision medicine” and at the same time speeding up the time of reporting. Furthermore the numerous data obtained by the AI analyses can provide in a short time several indications for the management of patients.

In CCTA, the application of AI is focused on detection of stenosis and analysis of plaques, however it is notable that information on the presence of ischemia can be obtained from rest images. Using this opportunity, the application of AI algorithms on CCTA could minimize the overestimation of pathology, providing a depth of information from a single examination data particularly for anatomy and potential ischemia.

In CMR, the application of AI is focused on solving delays in reporting time and the potential application on the reporting of examinations thoroughly completed in just a few steps.

The use of AI in the field of echocardiography has the potential to improve the diagnostic accuracy of cardiovascular disease, moving the interpretation of imaging data to a more advanced level, and helping in prognostic stratification while enhancing lab workflow.

The application of AI in clinical practice is rapidly growing. However, it is important to consider that AI algorithms may represent a helpful tool for the evaluation of images in cardiac imaging, allowing to speed up the reporting and interpretation [13, 121]. AI algorithms can not substitute human readers but they represent a useful tool for implementation of clinical workflow.

However, despite the methods in which the application of AI in cardiac imaging can be extremely helpful, several limitations need to be addressed. In particular, it is not negligible that all these algorithms need to be approved by the FDA or the European Community before their use in clinical practice. European Commission for application of AI in medicine suggested some rules regarding requirements on data collecting, analysis and transparency [13], furthermore it is important to evaluate carefully the data collected considering that development of an AI algorithm need a heterogeneous population that should not be unbalanced in terms of ethnicity or gender [13].

Furthermore it is important to consider that although many algorithms are available also as open source, the development of a robust algorithm needs training and validation on a large dataset. Therefore, vendors need a large amount of data in order to develop a reliable tool; the latter can be considered a limitation in terms of AI algorithms development.

Therefore, despite the bright future of cardiac imaging linked to the application of AI, it is important to consider the clinical safety of these algorithms should they be approved for use in clinical practice.

## Declarations

### Author contribution statement

All authors listed have significantly contributed to the development and the writing of this article.

### Funding statement

This research did not receive any specific grant from funding agencies in the public, commercial, or not-for-profit sectors.

### Data availability statement

No data was used for the research described in the article.

### Declaration of interests statement

The authors declare no conflict of interest.

### Additional information

No additional information is available for this paper.
